# Glanzmann Thrombasthenia Associated with Siderotic Synovitis and Arthropathy: A Case Report

**DOI:** 10.2147/JBM.S418937

**Published:** 2023-11-03

**Authors:** Mouhammad J Alawad, Mohammad Abu-Tineh, Awni Alshurafa, Alaa Al-Taie, Anil Yousaf, Mohamed A Yassin

**Affiliations:** 1 Department of Medical Education, Internal Medicine Residency Program, Hamad Medical Corporation, Doha, Qatar; 2 Department of Medical Oncology, Hematology and BMT Section, National Center for Hamad Medical Corporation, Doha, Qatar; 3 Clinical Imaging Department, Hamad Medical Corporation, Doha, Qatar

**Keywords:** Glanzmann thrombasthenia, hemarthrosis, synovitis

## Abstract

Glanzmann thrombasthenia is a bleeding disorder with a low incidence. It typically manifests as superficial bleeding episodes, which tend to be mild. Deep organ involvement is not uncommon but remains rare due to the rarity of the disease itself and the unusual association between platelet disorders and deep organ implications. A 17-year-old boy with Glanzmann thrombasthenia since infancy developed ankle pain after a minor trauma. His initial workup was negative, but he continued to experience ankle pain. A magnetic resonance imaging (MRI) done after four weeks suggested siderotic synovitis. The patient was lost to follow-up after that and returned after two years with recurrent left ankle pain. Imaging and studies have shown evidence of chronic arthropathy. A specialized orthopedic team assessed the patient. The patient underwent intra-articular steroid injection for pain relief and was referred to continue physical therapy. In conclusion, hemarthrosis is more common in hemophilia than in platelet disorders and has potential morbidity and quality-of-life implications.

## Introduction

Homeostasis is the process of blood clot formation at the site of a vessel injury. It consists of two distinct phases: formation of a platelet plug and clotting cascade activation, which leads to plug stabilization.^1^ Platelet activation is an important step that involves cell adhesion, aggregation, and secretion. Platelet aggregation is mediated by exposure to structural changes in the integrin alpha-IIb/beta-3 (αIIbβ3) receptor on the platelet surface, leading to the binding of von Willebrand Factor (VWF) and fibrinogen, resulting in platelet-platelet aggregation.^1^,^2^

Glanzmann thrombasthenia (GT) is a rare bleeding disorder caused by a mutant GPIIb/IIIa. It is inherited in an autosomal recessive fashion with an incidence of 1 in 1,000,000 patients.^3^ GT is mainly characterized by mucocutaneous bleeds, manifested as purpura, epistaxis, gingival hemorrhages, and menorrhagia. Deep organ involvement is well documented but remains sparse in the literature.^4^

Hemarthrosis is an infrequent and unusual condition associated with GT. However, it is a very common manifestation of hemophilia, with most affected patients in childhood.^4^,^5^ Herein, we present a case of GT in a young adult complicated with hemarthrosis after minor trauma, impacting mobility and physical activity, as reported by the patient. We present this case to highlight the rare association between hemarthrosis and GT and discuss the most convenient management plan for such cases.

## Case Presentation

A 17-year-old male was diagnosed with Glanzmann thrombasthenia for one year, and the diagnosis was based on recurrent epistaxis and gingival hemorrhage. He experienced epistaxis episodes almost every 3–4 months, for which he received a platelet transfusion. He was assessed for other bleeding disorders; his von Willebrand’s, factor VIII, and other clotting factor levels were all within normal limits, and his platelet count was within normal ranges. He had prolonged bleeding time; the platelet function analyzer (PFA) showed prolonged closure time with both collagen and epinephrine at 211 s (normal up to 175 s) and collagen and adenosine diphosphate (ADP) at 233 s (normal up to 116 s). Platelets failed to aggregate with ADP, arachidonic acid, or collagen. The only response observed was with ristocetin (1.5 mg/mL.

**Figure 1:**
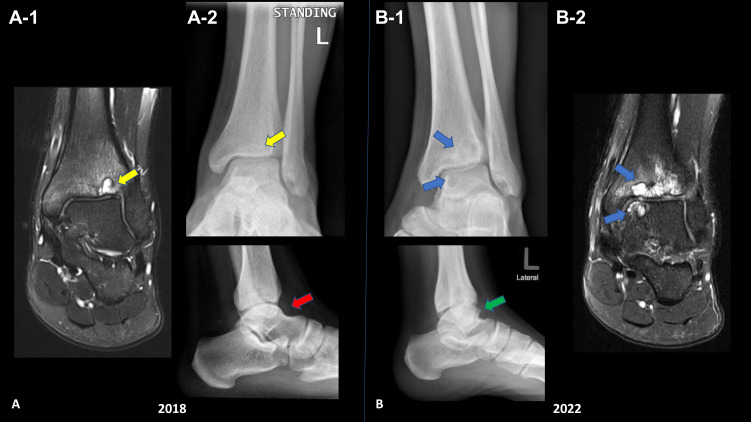
Comparative images: (**A**) Two sets of ankle radiographs (MRI A-1, Plain XR A-2) AP and lateral views first in 2018 showed mild ankle joint effusion (red arrow) and small tibial plafond subchondral cystic changes (yellow arrow). (**B**) 2022 follow-up radiographs (Plain XR B-1, MRI B-2) showed progressive changes with multiple large subchondral cysts involving the ankle joint opposing articular surfaces (blue arrows) as well as secondary osteoarthritis with osteophyte formation (green arrow) and reduced ankle joint space. Ankle MRI-selected images coronal PD FS are illustrating the progression of the articular pathology, increase the number of subchondral cysts.

In September 2018, the patient developed left ankle pain after twisting while playing sports; there was no direct trauma, swelling, or restricted joint movement. The patient visited a local health center. Physical examination was unremarkable without any effusion or other findings suggestive of hemarthrosis; labs results showed normal platelet count, prothrombin time (PT), and activated partial thromboplastin time (aPTT); plain ankle radiography did not show any definite significant bone or articular pathology (Figure 1A); depending on these findings, there was no suspicion of an acute injury like hemarthrosis, and the patient was discharged with pain killers.

Four weeks later, the patient returned due to pain and ankle discomfort. Ankle MRI showed 2018 mild ankle joint effusion and synovitis, with focal mass-like synovial proliferation noted at the anterior ankle recess, showing mild enhancement and blooming artifacts (Figure 1A). The radiological findings were likely indicative of siderotic synovitis. After MRI was performed, the patient did not attend the follow-up appointments.

In December 2021, he visited the emergency department because of left ankle pain after the injury. The pain occurs almost monthly for one or two days, and then resolves with rest and analgesia. Examination revealed no swelling or gross deformity, but some tenderness was observed. Magnetic resonance imaging (MRI) showed remarkable subarticular cystic changes in the tibial and talar aspects of the ankle joint, with overlying articular cartilage changes and synovial thickening mainly anteriorly.

**Figure 2:**
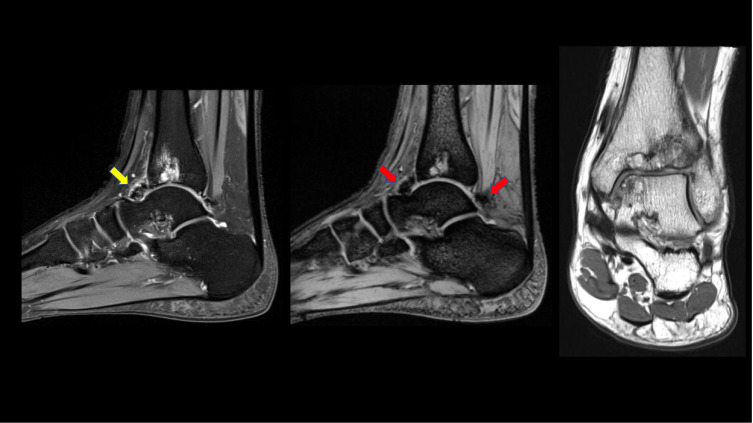
MRI of the ankle joint: sagittal T2WI, sagittal gradient sequence and coronal PDWI, show intra-articular synovial low signal intensity (yellow arrow) demonstrating blooming artifact on the gradient sequence (red arrows) in the anterior and posterior recess of the ankle joint consistent with blood degradation products related to repeated episodes of hemarthrosis. Again, secondary osteoarthritic changes are noted with multiple cystic changes/geodes with surrounding marrow oedema. Findings are consistent with siderotic chronic synovitis.

The patient was referred to an orthopedic clinic for review in February 2022. He reported that pain is usually bearable but increases with cold weather. No apparent swelling or tenderness was observed in the anterior aspect of the ankle. The images (Figure 1B) again demonstrated the same subarticular cystic-like changes with no interval changes. Owing to synovial changes, the patient was referred to a specialized ortho-oncology center for assessment in July 2022. Upon examination, the patient had a full range of motion, but experienced pain episodically. Magnetic resonance imaging (MRI) was performed during the visit. MRI findings were consistent with siderotic synovitis in the anterior and posterior ankle joint recesses, with a mild reduction in interval size anteriorly. Tibiotalar hemophilic arthropathy with opposing subarticular pseudocyst formation and chondromalacia was observed (Figure 2).

Due to the recurrent presentations of pain, the orthopedic team performed an intraarticular steroid injection in December 2022. Arthrocentesis did not yield any blood, and the procedure was uneventful. On follow-up in January 2023, the patient reported reasonable pain control and no joint swelling and was advised to continue a tailored physical therapy regimen.

## Discussion

Dr. Edward Glanzmann first described Glanzmann thrombasthenia in 1918, which is caused by a deficiency or abnormality of platelet glycoprotein IIB/IIIa, a surface protein that binds to either side of the fibrinogen molecule, allowing for platelet aggregation and the formation of a platelet plug at the site of the injury.^3^ In GT, this step does not occur properly; although the platelet count is generally normal, platelets cannot bind to fibrinogen and thus form a stable clot. The recognition of GT pathogenesis has improved our understanding of the role of platelets in homeostasis and their treatment and implantation.^1^,^3^

GT typically presents in infancy or early childhood. The diagnosis is suspected after bouts of bleeding, mainly after trauma, and the bleeding episodes are disproportionate to the bleeding occurring in a normal patient. When GT is suspected, other common bleeding disorders must be excluded.^3^,^4^ Initial laboratory assessment showed normal to near-normal platelet count and morphology, normal prothrombin time, activated partial thromboplastin time, prolonged bleeding time, and abnormal measurements of advanced platelet function tests, such as.^6^ Family history is essential, as GT’s incidence rate of GT is higher in certain ethnic groups due to consanguinity.^7^

Generally, bleeding due to platelet defects manifests as superficial bleeding symptoms and signs.^8^ In GT purpura and epistaxis are the most commonly encountered bleeds.^6^,^8^ Women are at high risk for menorrhagia and postpartum bleeding.^8^,^9^ The involvement of other organs has also been reported.^7^ In one study, hemarthrosis due to GT was quite rare; 3% of patients with GT had hemarthrosis,^5^,^10^ bearing in mind the low incidence of GT itself. Joint involvement is significant because it affects the patient’s functional status and quality of life.^11^

Based on the existing literature, hemarthrosis and arthropathy pathophysiology of GT do not differ from hemophilic arthropathy.^10^,^11^ Although both are bleeding disorders, their patho-physiologies are not similar. Siderotic arthropathy is a condition characterized by iron deposition in the synovium and joint tissues, leading to joint damage and dysfunction. It is commonly associated with chronic hemarthrosis, which involves recurrent bleeding in the joint space.^12^,^13^ The pathophysiological process of hemarthrosis involves the participation of inflammatory cells, interleukins, neo-angiogenesis, and the formation of a protein complex called inflammasome, which contributes to the inflammatory response and subsequent damage to the synovial membrane.^13^ This, in turn, induces synovial hypertrophy and fibrosis and, in the long run, loss of cartilage and development of subchondral cysts.^10^

There is no cure for GT, and the mainstay of management is platelet transfusion and anti-fibrinolytic agents if bleeding episodes occur or in preparation for an invasive procedure.^3^,^4^ However, hemarthrosis management depends on its presentation and severity. In acute cases with swelling and limited range of motion, aspiration of the synovial space to relieve effusion is used alongside joint rest and splinting if needed,^5^,^11^ whereas in long-standing insults such as in our case, physical therapy and rehabilitation regimens have shown some benefit in preserving and restoring functional activity,^11^,^14^ with care to avoid trauma and medications that could further impair platelet function such as NSAIDs and aspirin.^4^ A surgical approach may be needed with severe joint destruction and failure of other conservative measures.^11^ Our case is a rare entity that requires further studies through case reports, series, and reviews for a better understanding of the pathogenesis and course of the disease.

## Conclusion

GT is a rare bleeding disorder stemming from GPIIb/IIIa platelet dysfunction that typically presents with mucocutaneous bleeding. However, the unusual association between hemarthrosis and chronic arthropathy, as exemplified in this case, highlights the potential complications that can arise in this condition. Recurrent ankle pain, diagnosed as siderotic synovitis and tibiotalar hemophilic arthropathy, underscores the significance of early recognition and tailored management strategies to address these rare but impactful manifestations. As our understanding of Glanzmann thrombasthenia continues to expand, it is crucial to conduct further research, including case reports and studies, to deepen our insight into its pathogenesis and clinical course, ultimately enhancing the care and quality of life of individuals with this condition.

## Data Sharing Statement

Data and materials are available on request.
